# Cerebellar dysconnectivity in schizophrenia spectrum: task-based functional connectivity analysis and cognitive stratification

**DOI:** 10.3389/fpsyt.2026.1796599

**Published:** 2026-04-02

**Authors:** Diana S. M. Rosales-Gurmendi, Sadam Hussain, Eduardo de Avila-Armenta, Gerardo A. Fumagal-González, Jorge A. Garza-Abdala, Alma A. Pedro-Pérez, Jasiel Toscano, Jose G. Tamez-Peña

**Affiliations:** 1 School of Engineering and Sciences, Tecnologico de Monterrey, Monterrey, Nuevo Leon, Mexico; 2 School of Medicine and Health Sciences, Tecnologico de Monterrey, Monterrey, Nuevo Leon, Mexico; 3 School of Engineering, Pontificia Universidad Católica de Chile, Santiago, Chile

**Keywords:** cerebellum, cognitive subtypes, functional connectivity, schizophrenia, working memory

## Abstract

**Introduction:**

Schizophrenia is conceptualized as a disorder of brain network dysconnectivity, yet relationships between neural alterations, cognitive deficits, and genetic risk remain unclear.

**Methods:**

We examined 86 participants: schizophrenia patients (SCZ), unaffected siblings (SCZ-SIB), healthy controls (CON), and control siblings (CON-SIB). We used a multiscale graph-theoretic analysis of task-based fMRI during N-back working memory and unsupervised clinical-cognitive clustering.

**Results:**

We found that reduced cerebellum-sensorimotor (CER-SM) and cerebellum-cingulo-opercular (CER-CO) connectivity during the 1-back condition robustly discriminated SCZ from CON (AUC = 0.89). Critically, these dysconnectivity patterns were linked to clinical state, present in SCZ vs. SCZ-SIB but absent in SCZ-SIB vs. CON-SIB, suggesting illness expression rather than familial risk. Unsupervised clustering revealed three data-driven subtypes with distinct cognitive- symptomatic profiles: subtype 1 with relative preservation of verbal abilities (predominantly controls), subtype 2 with marked fluid cognitive impairment (enriched in SCZ), and subtype 3 with intermediate performance with working memory sparing (mixed composition). Cerebellar-cortical hypoconnectivity showed graded alignment across these profiles.

**Discussion:**

These findings demonstrate that cerebellar dysconnectivity is most detectable under moderate cognitive load, tracks with clinical state, and covaries with transdiagnostic cognitive profiles, advancing circuit-based understanding of schizophrenia heterogeneity.

## 1 Introduction

Schizophrenia is a disabling psychiatric condition marked by profound disruptions in perception, thought, and cognition Jauhar et al. (1). Beyond positive and negative psychotic symptoms, cognitive deficits, particularly severe and persistent impairment in working and episodic memory, are recognized as core features that largely determine functional outcome and show limited response to available antipsychotics Barch and Ceaser (2). Characterizing alterations in functional connectivity during cognitive tasks is therefore crucial for understanding the pathophysiology of schizophrenia. Neuroimaging research has established that schizophrenia involves altered functional connectivity patterns implicating high-order cerebral networks, including the default mode network, salience network, and executive control networks Sheffield and Barch (3), Anticevic et al. (4). However, most studies treat networks as homogeneous units and analyze connectivity exclusively at the macroscopic level, obscuring internal functional heterogeneity and preventing identification of specific regions responsible for observed alterations Diedrichsen et al. (5).

Additionally, traditional unimodal approaches analyze neuroimaging and clinical data in isolation, impeding identification of integrated translational subtypes that transcend diagnostic categories Honnorat et al. (6), Porter et al. (7). This limitation is particularly salient given recent advances in data-driven subtyping of schizophrenia, which demonstrate that neurobiologically distinct subgroups can be identified through unsupervised clustering of neuroimaging features, often transcending traditional symptom-based classifications Sheffield and Barch (3). While resting-state fMRI (rs-fMRI) has been widely employed to identify connectivity abnormalities, this approach faces significant limitations in schizophrenia research. Connectivity measured during rest may be confounded by uncontrolled differences in cognitive states between groups, such as systematic variations in internally directed cognition, rather than reflecting stable functional alterations per se Barch and Ceaser (2). Task-based paradigms constrain unregulated mental states, allowing more precise attribution of connectivity alterations to specific cognitive operations (Tan et al. (8), Uddin and Garavan (9)). In schizophrenia, working memory deficits represent a well-characterized endophenotype with established neural correlates Repovš and Barch (10). Examining functional connectivity during working memory performance allows direct investigation of how network coordination fails when the system is stressed by cognitive demand, a stress test approach that may reveal pathophysiological mechanisms invisible during passive rest Ji et al. (11).

The N-back paradigm is among the most versatile measures of working memory in cognitive neuroscience, inherently integrating encoding, maintenance, and retrieval processes while placing substantial demands on executive functions, including attentional control and online manipulation of information, precisely the domains most consistently impaired in schizophrenia Gilmour et al. (12). Importantly, the N-back permits parametric manipulation of cognitive load, enabling examination of how network connectivity scales with increasing memory and executive demands. Prior research demonstrates that functional connectivity within and between the fronto-parietal (FP), cingulo-opercular (CO), and cerebellar (CER) networks systematically increases with memory load in healthy individuals Pongpipat et al. (13). This load-dependent modulation is particularly relevant for evaluating cerebellar-cortical dysconnectivity, allowing us to test whether schizophrenia is characterized by (i) failure to appropriately recruit fronto-cerebellar circuits under moderate cognitive demands, (ii) progressive degradation of network integration with increasing load, or (iii) stable alterations independent of cognitive demand Ji et al. (11). The selection of N-back over other working memory paradigms was further motivated by its extensive validation in schizophrenia research and favorable characteristics for fMRI integration Gilmour et al. (12), Owen et al. (14). N-back performance reliably activates fronto-parietal-cerebellar circuits in healthy volunteers, with well-documented alterations in schizophrenia patients, including both hypofrontality and compensatory hyperactivation Huang et al. (15). The continuous nature of the task minimizes confounds related to performance differences that can complicate interpretation of block-design paradigms.

The cerebellum has emerged as a critical node in cognitive control circuits, extending far beyond its traditional motor functions to contribute to timing, sequencing, and error processing through extensive cortico-cerebello-thalamic pathways Schmahmann (16). In schizophrenia, converging evidence implicates cerebellar dysfunction in cognitive pathology. Recent meta-analytic and mega-analytic evidence confirms that cerebellar dysfunction is consistently implicated in schizophrenia pathophysiology, with structural and functional alterations in cerebellar Crus I-II and lobules VI-VIIIB associated with deficits in executive function, working memory, and cognitive flexibility Moberget et al. (17). Disruption of cerebellum-thalamo-cortical (CTC) circuits has been specifically linked to cognitive impairment associated with schizophrenia (CIAS), and hyperconnectivity in these pathways correlates with symptom severity and functional outcome Cao et al. (18). Notably, cerebellar-cortical connectivity appears particularly sensitive to cognitive state, with task-based connectivity revealing disorder-specific alterations not apparent at rest. Similarly, Tan et al. (8) identified distributed networks spanning the cortex, subcortex, and cerebellum that differentiate successful from unsuccessful working memory manipulation, with delayed recruitment of cerebellar and subcortical regions during incorrect trials suggesting that precise temporal coordination of these circuits is essential for cognitive performance.

Recent advances in computational psychiatry offer promising tools for overcoming these barriers Tusconi and Dursun (19), Wang et al. (20), demonstrating that graph-theoretic metrics are particularly well-suited for modeling the complex topology of FC data, enabling quantification of network organization at multiple scales, from individual nodes to global network properties Sunil et al. (21), Naderi et al. (22). When combined with unsupervised learning algorithms for data-driven phenotyping, these approaches enable the identification of latent clinical-cognitive subtypes and their associated neural signatures Wang et al. (20), Clementz et al. (23). Guided by recent evidence that cerebellar-cortical connectivity is particularly sensitive to cognitive state, we hypothesized that task-based functional connectivity during working memory performance would reveal alterations in schizophrenia that are most pronounced under moderate cognitive load. By examining patients with schizophrenia, their unaffected siblings, and healthy controls with and without familial risk, and integrating multiscale graph-theoretic analysis with data-driven clinical-cognitive stratification, this study aims to advance circuit-based understanding of schizophrenia pathophysiology.

This article is structured as follows. Section 2 describes in detail the methodology. Section 3 reports the results in order of objectives. Section 4 discusses the implications of these findings, methodological considerations, and future directions. Section 5 concludes with a synthesis of the main contributions and their translational relevance.

## 2 Materials and methods

**Figure 1:**
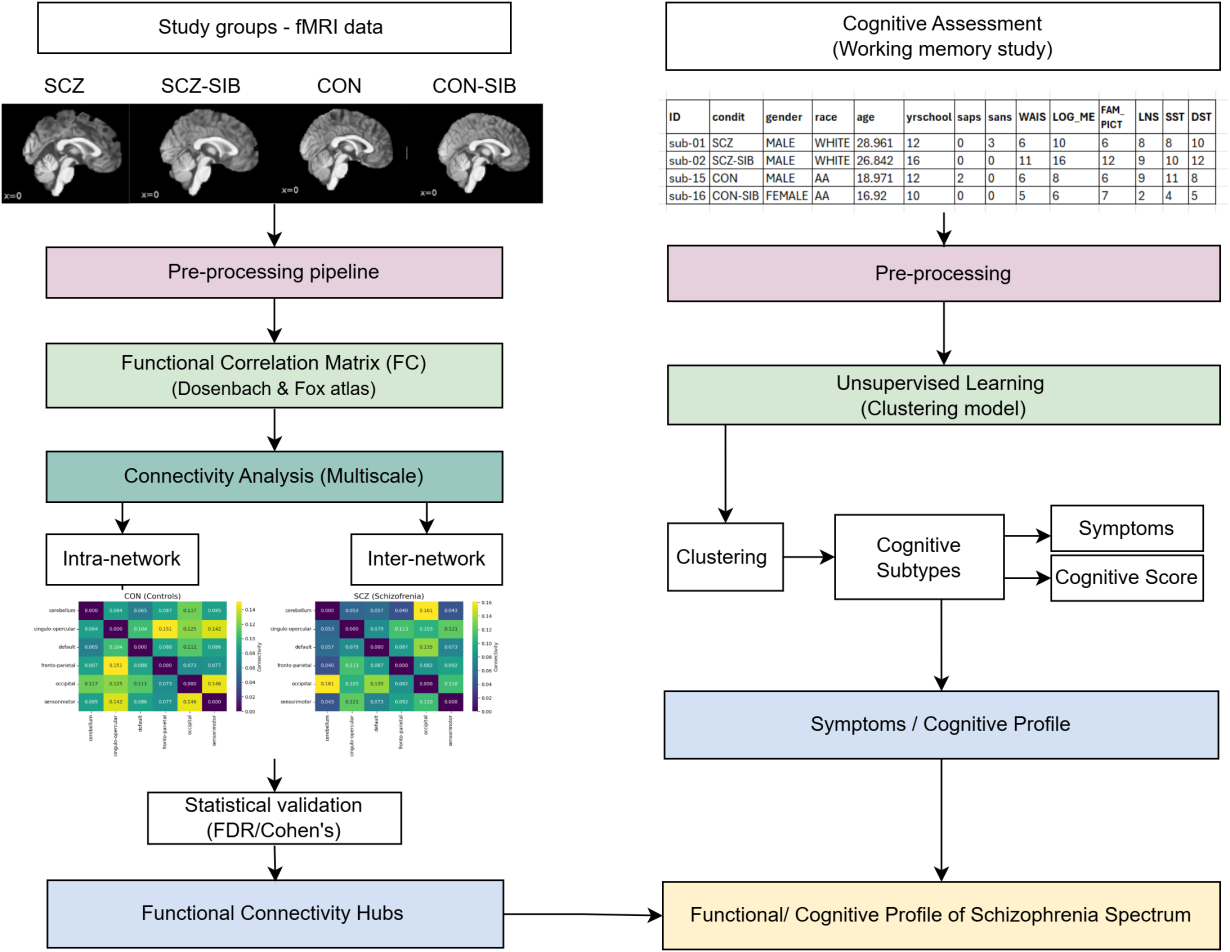
Analytical workflow for characterizing the schizophrenia spectrum. The workflow integrates two parallel streams: (left) functional MRI (fMRI) data from four study groups (SCZ, SCZ-SIB, CON, CON-SIB), processed to derive functional connectivity matrices, followed by inter- and intra-network analysis to identify connectivity hubs; and (right) clinical and cognitive data, preprocessed and subjected to unsupervised learning (clustering) to define clinical subtypes, which are then used to generate a cognitive profile. The final output is a unified profile integrating both neural and cognitive dimensions of the schizophrenia spectrum.

The analytical workflow, outlined in **Figure 1**, comprised the following phases: (1) preprocessing of images and clinical variables; (2) construction of functional brain networks using graph theory; (3) extraction of connectivity metrics at the nodal, inter- and intra-network level; (4) statistical analysis of differences between groups; and (5) unsupervised phenotypic stratification via consensus clustering.

### 2.1 Dataset

Data were taken from the publicly available Working Memory in Schizophrenia study Repovš and Barch (10) (https://openneuro.org/datasets/ds000115/versions/00001), originally collected by the Conte Center for the Neuroscience of Mental Disorders (CCNMD) at Washington University in St. Louis. All data were fully de-identified by the original investigators prior to public release. The original cohort comprised 98 participants: patients with DSM-IV schizophrenia (SCZ, n = 23), unaffected siblings of patients (SCZ-SIB, n = 34), healthy controls (CON, n = 20), and siblings of healthy controls (CON-SIB, n = 21). Importantly, this dataset contains exclusively task-based fMRI data and comprehensive clinical-cognitive assessments; no resting-state fMRI data are publicly available. This design feature, rather than representing a methodological limitation, provides an opportunity to examine how functional connectivity is organized under controlled cognitive demands, circumventing potential confounds associated with unregulated mental states during rest (see Introduction).

All participants underwent diagnostic evaluation through consensus between a research psychiatrist (semi-structured interview) and a trained research assistant (Structured Clinical Interview for DSM-IV Axis I Disorders) Kubler (24). As noted in the study Repovš and Barch (10), the patients with schizophrenia were outpatients with a mean illness duration of 4.79 years, clinically stabilized on antipsychotic medication.

See Repovs et al. Repovš and Barch (10). for at least two weeks (15 atypical antipsychotics; 4 combined typical/atypical regimens). Healthy controls had no lifetime history of Axis I psychotic or mood disorders and no first-degree relatives with psychotic illness. Siblings were full siblings based on self-report, with SCZ-SIB excluded for lifetime psychotic disorders but permitted non-psychotic comorbidities (major depression (21%), prior substance abuse (21%), prior substance dependence (7%), bipolar II disorder (7%), social phobia (7%), and PTSD (7%)). Similarly, siblings of healthy controls (CON-SIB) showed histories of prior substance abuse (24%), major depression (18%), prior substance dependence (6%), and social phobia (6%). All participants were excluded for substance dependence/severe abuse within 6 months, unstable medical conditions, head injury with neurological sequelae, or mental retardation.

Likewise, clinical assessments included structured diagnostic interviews, symptom severity (Scale for the Assessment of Positive Symptoms [SAPS] and Scale for the Assessment of Negative Symptoms [SANS]), and a comprehensive neuropsychological evaluation. Neurocognitive functioning was evaluated across four domains Repovsˇ and Barch (10): Working memory comprised Wechsler Memory Scale-III subtests (letter-number sequencing, digit span, spatial span), N-back task performance, and Continuous Performance Task d-prime scores. Episodic memory included Wechsler Memory Scale-III immediate recall subtests and California Verbal Learning Test free recall. Executive function encompassed Trail Making Test B, verbal and category fluency, WAIS-III matrix reasoning, and Wisconsin Card Sort perseverative errors. Intelligence (IQ) was estimated using WAIS/WASI vocabulary subtest scores. Demographic and clinical characteristics are summarized in **Table 1**.

**Table 1:** Demographic and clinical characteristics of the study cohort.

Measure	Group							
	Controls (CON)		Siblings of controls (CON-SIB)		Schizophrenia (SCZ)		Siblings of schizophrenia (SCZ-SIB)	
	Mean	SD	Mean	SD	Mean	SD	Mean	SD
Age (years)	20.6	5.1	21.1	4.4	24.2	3.7	24.1	3.5
Gender (% male)	60	—	61	—	72	—	58	—
Education (years)	12.3	3.7	12.3	3.3	12.1	1.8	13.5	2.3
Parental education (years)	15.0	1.6	14.6	1.4	14.5	2.4	14.9	2.5
Negative symptoms (score)	1.95	1.82	1.90	1.68	9.69*	4.06*	2.88	2.93
Positive symptoms (score)	0.25	0.71	0.05	0.22	4.78*	3.78*	0.47	0.74
IQ*^a^*	0.21	0.54	-0.42	0.76	-0.91*^a^*	0.67	-0.34	0.76
DST (score)	11.55	2.30	10.70	3.55	8.73	2.17	10.02	2.71
SST (score)	8.20	5.26	9.40	4.76	8.34	2.88	10.55	2.92
LNS (score)	11.05	2.94	10.90	2.95	8.21	2.41	9.79	2.89
Family picture (score)	9.55	3.01	10.25	2.42	6.73	3.22	9.14	2.38
Logical memory (score)	11.30	3.55	9.75	4.39	7.56	2.76	10.44	2.51
WAIS Matrics (score)	11.75	3.44	11.00	3.14	10.34	2.56	11.44	2.76
WAIS Vocabulary (score)	9.95	3.39	8.50	3.69	7.34	2.79	9.02	2.66

^*^p < 0.05 vs. CON, CON-SIB, and SCZ-SIB (ANOVA with Tukey HSD: SAPS *F* (3,93) = 31.92, *p <* 0.001, $\;\eta_{p}^{2}=0.51$; SANS F(3,93)=38.99, p<0.001, $\eta_{p}^{2}=0.56$).

*^a^*IQ scores reported as z-scores relative to the full sample.

#### 2.1.1 N-back working memory paradigm

Participants completed a working memory task with three load conditions: 0-back, 1-back, and 2-back. This parametric design enables systematic examination of how network connectivity scales with increasing cognitive demand, a critical feature given our hypothesis that cerebellar-cortical dysconnectivity would be most pronounced under moderate load (see Introduction). Each run consisted of two blocks per condition (0-back, 1-back, 2-back), with run order counterbalanced across subjects. Blocks followed a mixed block-event design: each began with a 2.5-second cue indicating the condition, followed by letters presented for 2.5 seconds each with variable inter-stimulus intervals (1 TR: 5%, 2 TR: 31%, 3 TR: 64%). Each block contained 21 trials (105 seconds total). Subjects indicated whether each letter matched: (a) a pre-specified target (0-back), (b) the immediately preceding letter (1-back), or (c) the letter two trials back (2-back). Three BOLD runs were acquired per participant (411 volumes total; 17.1 minutes scanning time) Repovš and Barch (10); see also Results.

#### 2.1.2 Data preprocessing

Data acquisition was performed at Washington University Medical School using a 3T Tim TRIO scanner (TR = 2500 ms, TE = 27 ms, voxel size = 4 × 4 × 4 mm). Functional images were preprocessed using fMRIPrep 20.2.1 Esteban et al. (25), followed by spatial smoothing (6-mm FWHM), high-pass temporal filtering (0.01 Hz), and intensity normalization to percent signal change. Quality control was performed via MRIQC and visual inspection Esteban et al. (26). Exclusion criteria included excessive motion (framewise displacement of 0.5 mm in 20% of volumes), non-standard spatial dimensions (any voxel array other than 64 x 64 x 64 x 137), or insufficient task duration. The final analyzed sample comprised 86 participants with valid data across all three task conditions (253 total graphs: 86 subjects × 3 tasks).

### 2.2 Functional connectivity construction

Functional networks were constructed using the Dosenbach et al. and Fox atlas Dosenbach et al. (27), comprising 165 regions of interest (ROI) assigned to six canonical networks: Default Mode (DMN), Fronto-Parietal (FP), Cingulo-Opercular (CO), Sensorimotor (SM), Occipital (OCC), and Cerebellar (CER). Consistent with the baseline study Repovš and Barch (10), for each participant (*s*) and N-back condition (*t*) (0-back, 1-back, 2-back), mean BOLD time series were extracted from every ROI. Pairwise Pearson correlation coefficients yielded symmetric 165 x 165 connectivity matrices $A_{s}^{\left(t\right)}\in {\mathbb{R}}^{165\times 165}$ normalized via Fisher’s r-to-z transformation. To preserve network structure while reducing noise, matrices were thresholded at the 85th percentile of absolute off-diagonal values, yielding sparse weighted graphs. Diagonals were zeroed and matrices symmetrized, yielding 253 valid graphs (86 subjects x 3 tasks).

Graph-theoretic metrics were extracted at the nodal and network levels using NetworkX 3.1 and Nilearn 0.10.1:

• Nodal-level: For each ROI (*i*), we computed its weighted degree (strength) as the sum of the absolute weights of its connections, $deg_{i}=\sum_{j}|A_{ij}|$, and local clustering coefficient *C_i_*.

• Network-level: ROIs were grouped by canonical network assignments. We computed the following: Inter-network connectivity (Equation 1) as the mean weight of pairwise inter-ROI connections between networks (k) and (m):

(1) $$Inter_{km}^{\left(s,t\right)}=\frac{1}{\left|N_{k}|\cdot |N_{m}\right|}\sum_{i\in N_{k}}\sum_{j\in N_{m}}A_{ij}^{\left(s,t\right)}.$$

Intra-network connectivity (mean weight of intra-network edges within network *k*):

(2) $$Intra_{k}^{\left(s,t\right)}=\frac{1}{\left|N_{k}|(|N_{m}|-1\right)}\sum_{i\in N_{k}}\sum_{j>i}A_{ij}^{\left(s,t\right)},$$

and Network degree as the sum of all inter-network connectivity values for a network *k*, representing total integration with the rest of the connectome.

Both metrics were calculated separately for the 0-back, 1-back, and 2-back conditions. This resulted in a compact, interpretable feature set of 21 inter-network links, 6 intra-network values, and 6 network degree values (33 features total) per subject and task, serving as input for group comparison and association analyses.

### 2.3 Statistical validation

The study employs statistical techniques that address limitations of traditional ANOVA-based approaches. We adopt robust, assumption-free tests and apply stringent corrections for multiple comparisons to enhance the reliability of inferences regarding group differences in functional network organization. Group comparisons employed Welch’s independent two-sample t-tests, accommodating unequal variances via the Welch-Satterthwaite equation. This test is defined as:

(3) $$t=\frac{{\bar{X}}_{1}-{\bar{X}}_{2}}{\sqrt{\frac{s_{1}^{2}}{n_{1}}}+\sqrt{\frac{s_{2}^{2}}{n_{2}}}},$$

were ${\bar{X}}_{1}$ and ${\bar{X}}_{2}$ are the sample means, $s_{1}^{2}$ and $s_{2}^{2}$ are the sample variances, and $n_{1}$ and $n_{2}$ are the sample sizes for groups 1 and 2, respectively. The degrees of freedom ( ν) are approximated using the Welch-Satterthwaite equation:

(4) $$\nu \approx \frac{{\left(\frac{s_{1}^{2}}{n_{1}}+\frac{s_{2}^{2}}{n_{2}}\right)}^{2}}{\frac{{\left(s_{1}^{2}/n_{1}\right)}^{2}}{n_{1}-1}+\frac{{\left(s_{2}^{2}/n_{2}\right)}^{2}}{n_{2}-1}}.$$

This approach is particularly suited for neuroimaging data, where homogeneity of variance cannot be assumed, providing a more conservative and valid estimate of significance compared to the standard Student’s t-test.

To control the false discovery rate (FDR) across the high number of comparisons inherent in connectome-wide analysis, we applied the Benjamini-Hochberg procedure to the p-values from each family of tests (e.g., all inter-network pairs within a task condition). We declare a result statistically significant if its FDR q-value is below the threshold of *q <* 0.05. Effect sizes were quantified using Cohen’s *d* for standardized mean difference, interpreting effect as small ( |d|≈0.2), medium ( |d|≈0.5), or large ( |d|≥0.8). This dual-threshold framework (statistical significance and biological relevance) ensures robust statistical validation.

#### 2.3.1 Familial validation

To dissociate clinical state from genetic risk, we implemented three neurobiologically interpretable contrasts: First, a primary case-control model compares individuals with schizophrenia (SCZ) against demographically matched healthy controls (CON) separately for each of the three N-back task conditions (0-back, 1-back, 2-back). This establishes the basic phenotype of illness-related dysconnectivity. Second, to dissociate the effects of clinical state from genetic risk, we implement an extended family-based model. This model includes all four study groups: schizophrenia patients (SCZ), their non-psychotic siblings (SCZ-SIB), healthy controls (CON), and the siblings of controls (CON-SIB). This design enables three specific, neurobiologically interpretable contrasts: SCZ vs. CON, which tests the effect of the manifest illness; SCZ vs. SCZ-SIB, which tests the effect of the clinical state within a shared genetic and familial environment; and SCZ-SIB vs. CON-SIB, which tests the effect of genetic liability in the absence of overt psychosis. Analyses were conducted using Python 3.11 (SciPy and Statsmodels libraries). Complete statistical results, including t-values, p-values, FDR q-values, and Cohen’s *d*, are available in **Supplementary Materials**.

#### 2.3.2 ROC analysis

To evaluate the individual-level discriminative power of connectivity findings, we performed a receiver operating characteristic (ROC) analysis using a composite score derived from significant network-level metrics. For each task condition, we identified inter-network connectivity metrics showing significant group differences (FDR *q <* 0.05). For each significant metric *j*, we extracted the absolute Cohen’s (*d*) effect size |*d_j_*| as a weight. For each subject *i*, the composite discriminant score was calculated as follows:

(5) $$CompositeScore_{i}=\sum_{j}(Connectivity_{ij}\times |d_{j}|),$$

where *Connectivity_ij_*, is the subject’s value for the metric *j* (e.g., CER-SM connectivity strength). This weighting |*d_j_*| ensures that metrics showing larger between-group effects contribute more to the final score, theoretically enhancing its classification power. The composite score served as a univariate input for binary classification (SCZ vs. CON). We generated the ROC curve and calculated the area under the curve (AUC) with 95% confidence intervals. The optimal threshold was determined using the Youden Index, from which sensitivity and specificity were derived.

### 2.4 Unsupervised phenotypic stratification

To identify clinical-cognitive subtypes transcending diagnostic categories, we performed unsupervised clustering on multidimensional clinical and cognitive data. The feature vector comprised global SAPS and SANS scores. The SAPS consists of 34 items rated 0–5 and assesses four domains (hallucinations, delusions, bizarre behavior, and positive formal thought disorder), whereas the SANS contains 25 items rated on the same scale and evaluates five domains (affective flattening, alogia, avolition–apathy, anhedonia–asociality, and attention). Cognitive performance was evaluated with a standardized neuropsychological battery, Wechsler (28), that included the following tests: Verbal comprehension (vocabulary subtest of the Wechsler Adult Intelligence Scale-III); processing speed and visual attention (Symbol Search and Trail Making Test Part A); episodic verbal memory (Logical Memory I and II from the Wechsler Memory Scale); visual recognition memory (Family Pictures I and II); and working memory (letter-number sequencing (LNS) and digit span (forward and backward) subtests). To enable direct comparison across measures with different native scales, scores were min-max normalized to [0,1], as shown in the following equation:

(6) $$x_{norm}=\frac{x-x_{min}}{x_{max}-x_{min}},$$

where *x*_min_ and *x*_max_ represent the theoretical minimum and maximum values of each scale (e.g., 0–5 for SAPS/SANS items; observed range for raw cognitive scores). For variables with missing values (e.g., incomplete neuropsychological test results or missing SAPS/SANS domain scores), we employed listwise deletion, excluding subjects with any missing data points from analyses requiring complete cases. This approach was chosen to preserve the integrity of the multivariate clustering analysis and avoid potential bias introduced by imputation. The final cohort included in the clustering analysis (n = 98) reflects participants with fully complete clinical-cognitive profiles.

To identify biologically meaningful subgroups that transcend traditional diagnostic categories, we performed unsupervised clustering on the multidimensional clinical-cognitive data. The feature vector for each participant comprised the normalized global scores of the SAPS and SANS, the five domain scores from each scale, and the nine normalized cognitive variables described above, totaling 19 features. Clustering was performed using the Evacluster R package, Naderi et al. (22), an unsupervised clustering framework designed for discovering disease subtypes through consensus analysis and stochastic evaluation Nezhadmoghadam et al. (29). This approach incorporated Partitioning Around Medoids (PAM) with UMAP dimensionality reduction. The analysis ran 100 iterations for consensus matrix generation. Cluster stability was evaluated via Proportion of Ambiguous Clustering (PAC), Rand Index, Jaccard similarity, silhouette width, and Clinski-Harabasz index. Non-random partition was confirmed via permutation testing (10,000 shuffles, *p <* 0.001). This data-driven approach enabled identification of latent subtypes characterized by distinct combinations of symptom score and neurocognitive performance, independent of DSM-based labels. **Figure 1** illustrates the analytical workflow integrating neuroimaging and clinical-cognitive streams.

## 3 Results

N-back data was analyzed using a multiscale analysis and dual-threshold validation. Load (0-back, 1-back, 2-back) served as a within-subject factor, while genetic liability (SCZ or CON) and family member type served as between-subject factors. Graph-theoretic analysis of functional connectivity matrices revealed that cerebellar-cortical dysconnectivity emerged most prominently during moderate cognitive demand (1-back condition). The 1-back working memory condition served as the optimal probe, unmasking robust and replicable alterations in large-scale network communication that survived the false discovery rate (FDR) correction. In contrast, effects observed during the 0-back and 2-back conditions were either statistically nonsignificant after correction or showed divergent patterns, indicating that core network pathophysiology is most detectable under moderate, sustained cognitive load.

### 3.1 Inter-network connectivity

**Figure 2:**
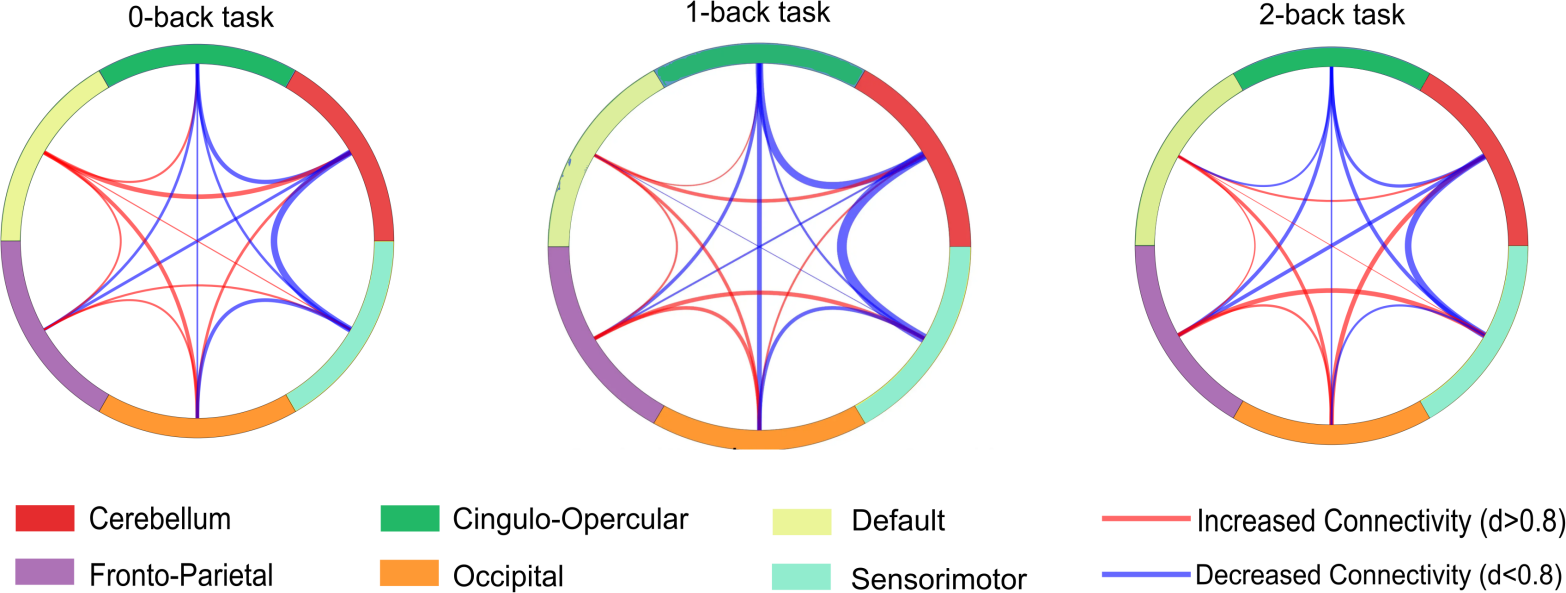
Functional connectivity differences between Schizophrenia (SCZ) and Healthy Controls (CON) across working memory tasks. Circular chord diagrams illustrate inter-network connectivity for the 0-back, 1-back, and 2-back working memory tasks. The visual representation highlights the pattern of altered connectivity (red = increased, blue = decreased) that varies as a function of task difficulty. The six functional networks are color-coded: Cerebellum (red), Cingulo-Opercular (green), Default (yellow), Fronto-Parietal (purple), Occipital (orange), and Sensorimotor (turquoise).

**Figure 2** illustrates inter-network connectivity across working memory loads. Visual inspection reveals distinct patterns of altered connectivity (red = increased, blue = decreased) as a function of task difficulty. The analysis identified a significant reduction in CER-SM connectivity (quantified using Equation 1) in the SCZ group compared to healthy controls (CON) as the most reliable result. In the 0-back task condition, we observed that connectivity between the cerebellum (CER) and sensorimotor (SM) networks was reduced (indirect effect size, *d* = −1.078) and moderate increase between CER and default mode network (DMN) connectivity (small effect size, *d* = 0.726). However, these effects were not significant following the strict false discovery rate (FDR) significance threshold (*q >* 0.05). While observable across all tasks, the most robust effects emerged during the 1-back condition. Here, the reduction in CER-SM connectivity was statistically significant (Equation 3; CER-SM: Cohen’s *d* = −1.547, FDR *q* = 0.0015) with degrees of freedom approximated via Equation 4 to accommodate unequal variances. A second significant finding was a reduction in CER-CO connectivity (CER-CO: Cohen’s *d* = −1.155, FDR *q* = 0.0298). No other inter-network pairs showed significant group differences after FDR correction across any task condition. Under high cognitive load (2-back), the CER-SM reduction remained large (CER-SM: *d* = −0.914, *q* = 0.28) but was not significant. At the intra-network level in the 2-back condition, we found reductions in the clustering coefficient of the DMN (assessed via Equation 2) (small effect size, *d* = −0.711) and in both the degree (small effect size, *d* = −0.858) of the SM network, though these were also not significant after FDR analysis.

**Figure 3:**
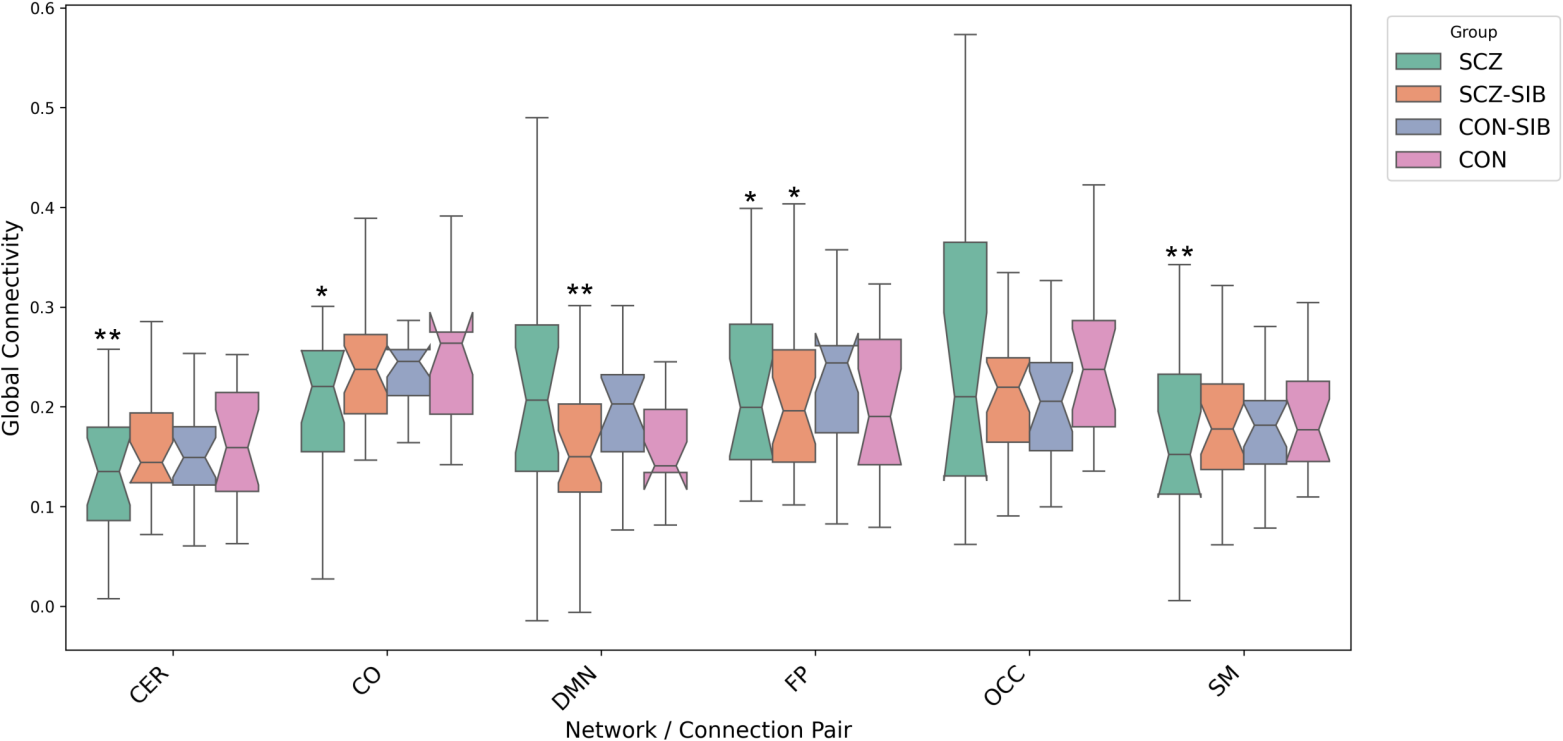
Intra-network global connectivity during the 1-back task condition. Boxplots show the distribution of global connectivity for each network across schizophrenia patients (SCZ), their nonpsychotic siblings (SCZ-SIB), healthy controls (CON), and control siblings (CON-SIB). This comparison highlights the association of reduced cerebellar (CER) network connectivity with the clinical state of illness. *p < 0.05 **p < 0.01.

To determine whether observed dysconnectivity reflects clinical state versus genetic risk, we conducted three planned contrasts using the 1-back condition (where effects were maximal). **Figure 3** displays intra-network global connectivity distribution across all four groups.

Clinical state (SCZ vs. SCZ-SIB): SCZ patients exhibited significantly lower CER-SM connectivity than their non-psychotic siblings (*d* = −1.086), accompanied by reduced intra-network organization of the cingulo-opercular network (decreased degree and clustering, *q <* 0.01). Genetic Risk (SCZ-SIB vs. CON-SIB): In stark contrast, no significant differences emerged in any inter- or intra-network metric between non-psychotic siblings of patients and siblings of controls across all task conditions (*q >* 0.05). Familial Environment (CON vs. CON-SIB): Similarly, no significant differences were observed between healthy controls and their siblings, ruling out non-specific effects of shared family environment on network connectivity.

### 3.2 Intra-network organization and task modulation

**Figure 4:**
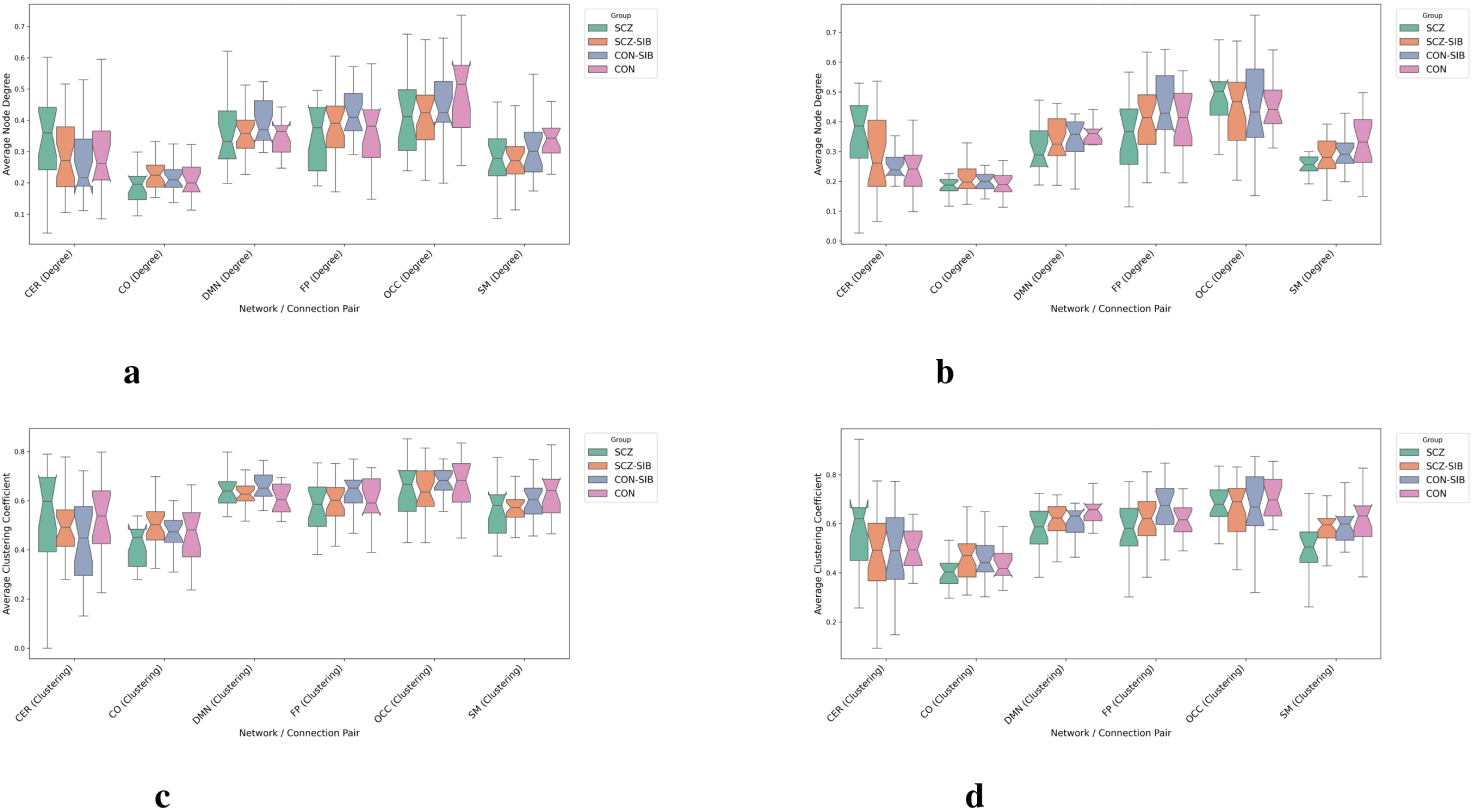
Task-dependent alterations in intra-network topology in schizophrenia. Boxplots depict the distribution of **(a, b)** average node degree and **(c, d)** average clustering coefficient for each network across four groups. Panels **(a)** and **(c)** show data from the 1-back condition; panels **(b)** and **(d)** from the 2-back condition. These metrics quantify the intrinsic organization of each network and its modulation by cognitive demand.

**Figure 4** illustrates task-dependent alterations in intra-network topology. Beyond the clinical-state effects in CO network organization described above, the sensorimotor (SM) network showed intrinsic disruptions in SCZ patients: the average degree was reduced in both the 1-back (*d* = −0.683) and 2-back (*d* = −0.906) conditions, and its local clustering was impaired in the 2-back condition (*d* = −0.858). Notably, SM network disorganization intensified with increasing cognitive load, suggesting load-dependent degradation of intrinsic network architecture in schizophrenia. The default mode network (DMN) showed a reduced clustering coefficient during 2-back (*d* = −0.711), though this did not survive FDR correction. No significant intra-network alterations were observed in FP or OCC networks.

#### 3.2.1 ROI-level analysis: identification of dysfunctional hubs

Multiscale analysis localized network-level dysconnectivity to specific anatomical hubs by the ROI-level analysis. One finding transcended all cognitive loads: the inferior cerebellum consistently emerged as the most dysfunctional hub in the SCZ vs. CON group, showing reduced nodal degree across all task conditions (*q <* 0.001). This anatomical specificity transcended task demands, indicating stable cerebellar pathology. Furthermore, default mode network hubs showed reduced connectivity, including the angular gyrus (*d* = 1.073), precuneus (*d* = 0.584), and intraparietal lobule (*d* = 0.888) during the 1-back condition, extending to the posterior cingulate cortex at higher loads (*d* = 0.567). Conversely, a potential, task-specific compensatory occipital response was observed in the posterior occipital cortex, which exhibited increased nodal degree specifically during the low-demand cognitive (0-back: *q <* 0.001). These ROI-level findings converge with the network-level results, reinforcing the model of a specific breakdown in cerebellar-cortical and DMN circuitry associated with schizophrenia spectrum.

#### 3.2.2 Individual-level discriminative power of network biomarkers

**Figure 5:**
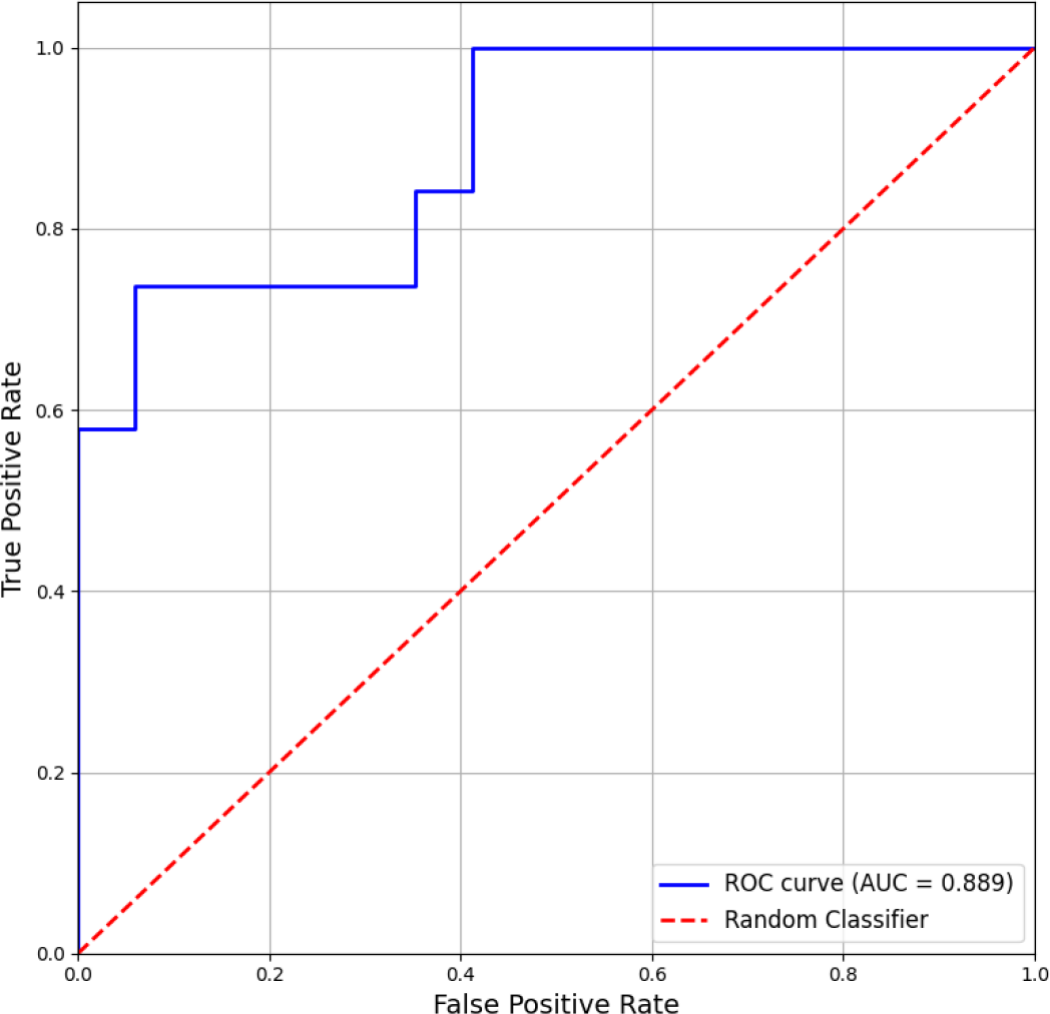
Predictive validation using a composite score based on statistically significant functional connectivity metrics. The ROC curve illustrates the ability to classify individuals with schizophrenia (SCZ) versus healthy controls (CON) during the 1-back working memory task. The resulting AUC of 0.889 demonstrates that these statistical findings translate into a robust predictive model.

As specified in our analytical approach, we conducted receiver operating characteristics (ROC) analysis to validate that functional connectivity during the 1-back condition provides superior individual-level discrimination between schizophrenia patients and healthy controls compared to other task loads. Based on the FDR-significant connectivity metrics identified in Section 3.1, we constructed a weighted composite score (**Equation 5**) using the two 1-back inter-network connections that showed robust group differences (N = 43; *SCZ* = 23*,CON* = 20): cerebellum-sensorimotor (CER-SM: *q* = 0.0015, *d* = −1.547) and cerebellum-cingulo-opercular (CER-CO: *q* = 0.0298, *d* = −1.155). The weighting scheme ensures that metrics with larger between-group effect sizes contribute proportionally more to the discriminant function. **Figure 5** presents the ROC curve for the 1-back composite score, showing an area under the curve (*AUC* = 0.889, 95% CI: 0.81-0.97). At the optimal threshold determined by the Youden Index, classification accuracy showed a sensitivity of 84% and a specificity of 82%. A *post-hoc* analysis of misclassified cases from the 1-back task was performed to characterize the model’s limitations. Of the 23 SCZ patients, four (17,4%) were misclassified as controls. These misclassified individuals were more likely to belong to the intermediate cognitive subtype (3 of 4, 75%) than correctly classified patients (8 of 19, 42%; Fisher’s test, *p* = 0.042). They also exhibited milder negative symptoms (SANS total score: 38.2 ± 5.1 vs. 48.7 ± 6.3) and higher working memory performance (LNS score: 0.65 ± 0.1 vs. 0.42 ± 0.2).

### 3.3 Unsupervised phenotype stratification

**Figure 6:**
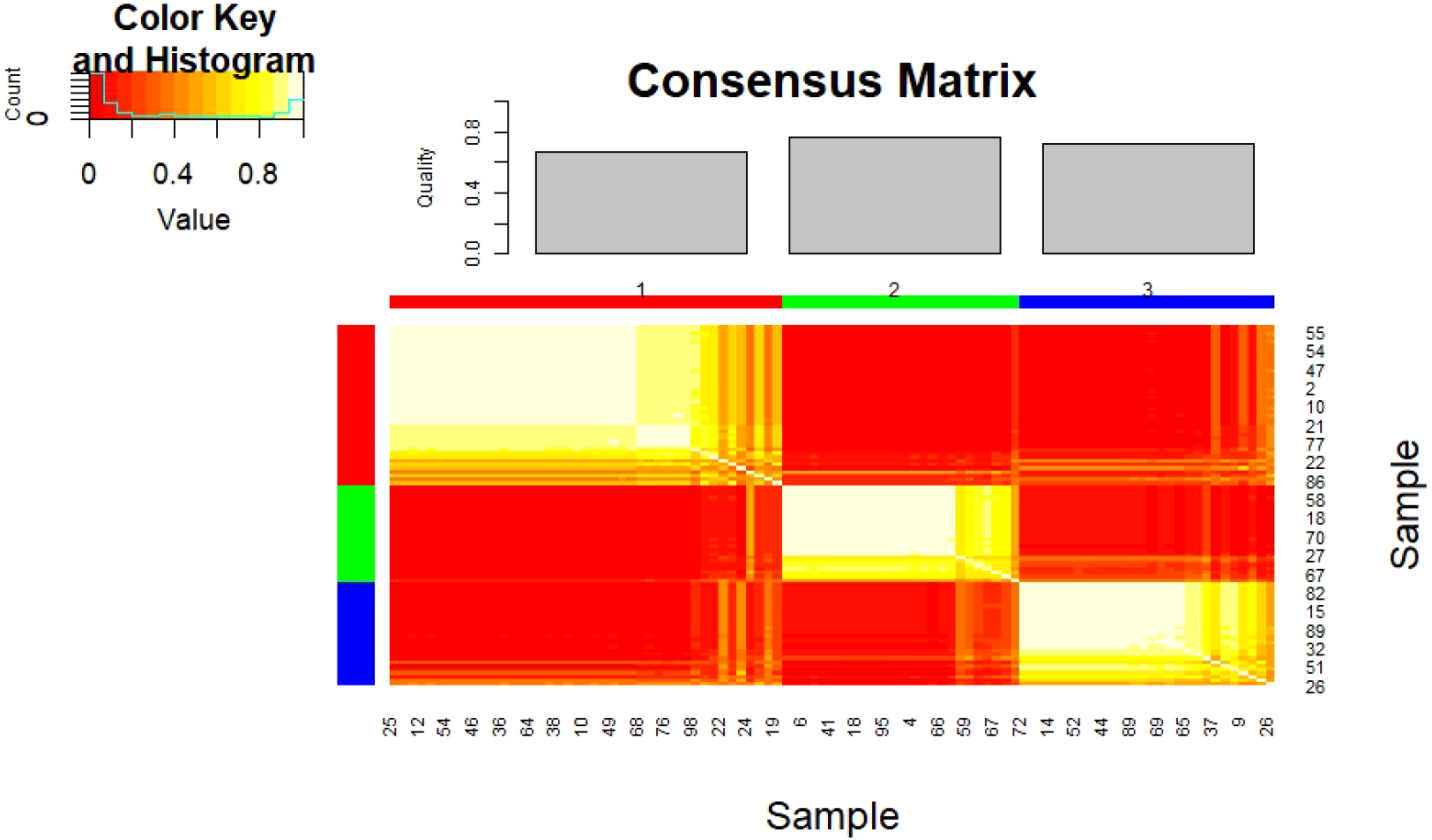
Consensus matrix illustrating the stability of the three identified clusters. Each cell represents the probability (0–1) that two subjects are assigned to the same cluster across multiple clustering iterations.

**Figure 7:**
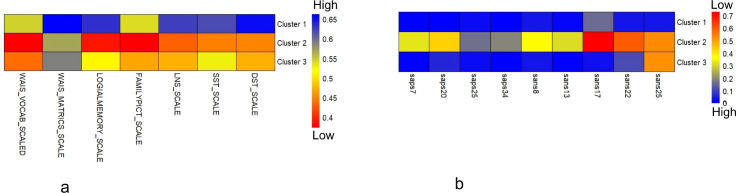
Cognitive and symptom profiles of data-driven patient clusters. Heatmaps depict mean standardized scores for seven cognitive tests (left) and nine SAPS/SANS symptom scales (right). Warmer colors denote lower cognitive performance (left) and greater symptom score (right).

Consensus clustering of clinical-cognitive data (19 features: SAPS/SANS domains, neuropsychological scores; all normalized via **Equation 6**) identified three stable subtypes in the full cohort (n = 98), confirmed by high consensus matrix stability (**Figure 6**), significant silhouette width, Calinski-Harabasz index, and permutation testing (10,000 shuffles, permutation *p <* 0.001). The consensus matrix displays high within-cluster agreement (red blocks) and low between-cluster overlap (yellow/white off-diagonal regions), indicating robust partition quality. **Figure 7** presents standardized cognitive and symptom profiles across subtypes. Panel (a) shows cognitive performance (higher values = better performance; blue = high, red = low); Panel (b) shows symptom score (higher values = greater severity; blue = low, red = high).

Subtype 1 (n = 36, 56% CON, 44% CON-SIB) comprised predominantly healthy controls and siblings with high performance across WAIS-III Vocabulary and WMS Family Pictures (0.60–0.65) with minimal SAPS/SANS burden across all domains (0.00–0.10). Subtype 2 (n = 29, 79% SCZ, 21% SCZ-SIB) marked impairment in WAIS-III Vocabulary (0.35–0.40), WMS Logical Memory (0.40–0.45), and LNS/Digit Span (0.45–0.50) with elevated SANS scores (affective flattening (0.60–0.70), avolition (0.50–0.60), and anhedonia-asociality (0.40–0.50)), and SAPS Hallucinations (0.40–0.50), Delusions (0.30–0.40), Bizarre Behavior (0.20–0.30), and Positive Formal Thought Disorder (0.30–0.40). Subtype 3 (n = 33, 58% SCZ/SCZ-SIB, 42% CON/CON-SIB) demonstrated moderate overall cognitive performance with relative preservation of working memory measures (0.50–0.55), despite heterogeneous diagnostic composition. High stability metrics (silhouette width, Calinski-Harabasz) confirmed these as robust data-driven partitions.

## 4 Discussion

This study integrates multiscale graph-theoretical analysis of task-based fMRI with unsupervised clinical-cognitive clustering to characterize neural and clinical heterogeneity in schizophrenia. We identified reproducible cerebellum-sensorimotor (CER-SM) and cerebellum-cingulo-opercular (CER-CO) hypoconnectivity, with maximal discrimination achieved under moderate working memory load (1-back). This task-optimized detection reflects optimal engagement of cerebellar-cortical circuits without the floor effects of higher loads or insufficient challenge of lower loads. Independently, data-driven clustering revealed transdiagnostic subtypes stratified by cognitive performance and symptom score, enabling examination of neural-behavioral convergence.

According to Chen and Desmond (30), verbal working memory engages distinct fronto-cerebellar systems for articulatory rehearsal and phonological storage, with moderate load optimally activating these circuits. Hudgins et al. (31) demonstrated that functional connectivity within fronto-parietal, cingulo-opercular, and cerebellar networks systematically increases with memory load in healthy individuals, suggesting that schizophrenia disrupts this parametric modulation. The 1-back condition thus represents an optimal challenge point where cerebellar-cortical coordination is sufficiently engaged to reveal pathophysiological alterations, consistent with Tan et al. (8) finding that delayed recruitment of cerebellar-subcortical regions characterizes unsuccessful working memory manipulation. This aligns with the hypothesis that schizophrenia involves disrupted timing and coordination across cortico-cerebellar networks, a pattern termed “cognitive dysmetria” (Cao and Cannon (32)). The absence of significant group differences at 0-back and 2-back likely reflects distinct constraints: minimal load insufficiently challenges cerebellar-cortical circuits, while maximal load induces performance homogenization that reduces discriminative variance.

The four-group design enabled examination of whether cerebellar dysconnectivity reflects manifest illness, genetic liability, or shared familial environment. The presence of significant CER-SM and CER-CO hypoconnectivity in schizophrenia patients compared to their unaffected siblings, coupled with the absence of differences between sibling groups, suggests that these alterations track with clinical state rather than constituting stable endophenotypes. This pattern is consistent with recent meta-analytic evidence indicating that cerebellar structural and functional abnormalities demonstrate high consistency across the lifespan, detectable at disease onset (Moberget et al. (17)). However, the large effect sizes for CER-SM hypoconnectivity across all task conditions (0-back:*d* = −1.078; 1-back:*d* = −1.547; 2-back:*d* = −0.914) indicates that circuit alterations are present but variably detectable, aligning with dimensional frameworks proposing that biomarkers reflect graded dysfunction Clementz et al. (23).

The identified circuits align with distinct functional architectures implicated in schizophrenia pathophysiology. Cerebellar-sensorimotor (CER-SM) dysconnectivity may reflect disruption of sensorimotor prediction and temporal coordination mechanisms fundamental to cognitive processing. The cerebellum’s role in forward modeling extends to cognitive domains through cortico-cerebellar-thalamic pathways Diedrichsen et al. (5), with deficient CER-SM connectivity potentially impairing temporal precision of neural responses. This interpretation is supported by Hudgins et al. (31), who demonstrated transdiagnostic association between cerebellar-subcortical connectivity and symptom assessments across schizophrenia, bipolar disorder, and ADHD. Cerebellar-cingulo-opercular (CER-CO) dysconnectivity implicates circuits critical for task-set maintenance and performance monitoring. The cingulo-opercular network supports stable task engagement and error processing, while the cerebellum contributes to error correction through cerebellar nuclei-thalamic-prefrontal pathways (Ide and Li (33)). Disrupted CER-CO connectivity may impair cognitive set maintenance, consistent with observations of perseverative errors in schizophrenia. The co-occurrence of CER-SM and CER-CO dysconnectivity suggests distributed cerebellar system dysfunction affecting both sensorimotor prediction and cognitive control, aligning with the “universal cerebellar transform hypothesis” (Diedrichsen et al. (5), Wolff and Northoff (34)).

Unsupervised clustering of clinical and cognitive variables identified three stable subtypes characterized by distinct patterns of performance across the administered assessment battery. This approach was motivated by the recognition that multivariate configurations of cognitive and symptomatic measures may reveal structure not apparent through univariate inspection or categorical diagnosis alone, consistent with contemporary evidence for cognitive heterogeneity in psychosis spectrum conditions Zhang et al. (35). The stratification revealed distinct performance profiles: Subtype 1 exhibited higher relative performance across WAIS-III Vocabulary and WMS (Logical Memory and Family Pictures), alongside minimal SAPS/SANS scores. The concentration of healthy individuals and unaffected siblings in this cluster is consistent with the expected distribution of these measures in non-clinical populations, though this pattern does not establish “preserved” function in absolute terms Karcher et al. (36), only relative to the present sample’s range.

Subtype 2 showed marked impairment in fluid cognitive domains, working memory (letter-number sequencing, digit span), logical memory, and the WAIS test, with elevated negative symptoms and relative sparing of crystallized verbal abilities, a dissociation consistent with established frameworks in schizophrenia Karcher et al. (36), Saleh et al. (37). Subtype 3 demonstrated intermediate performance with relative preservation of working memory measures, suggesting heterogeneity in cognitive deficit profiles. Notably, cerebellar dysconnectivity showed graded alignment with cluster membership: most pronounced in Subtype 2, intermediate in Subtype 3, and minimal in Subtype 1. These subtypes were derived from normalized scores relative to the present sample, not population-normed impairment. The cross-sectional design precludes classification of clusters as stable traits, illness stages, or compensatory states, and the relative preservation of specific working memory components in Subtype 3 requires replication with broader cognitive batteries. The methodological decision to exclude neuroimaging features from clustering was deliberate, enabling independent validation of whether neural signatures converge with behaviorally derived subtypes. This approach follows recommendations for multivariate phenotypic characterization preceding neural association studies (Uddin and Garavan (9)). Future research should test whether fMRI signatures prospectively predict cluster membership, enabling truly integrated biomarker-stratified models.

This study has several limitations. The cross-sectional design precludes causal inferences regarding development trajectories or progression risk. All patients were stabilized on antipsychotic medication, which may modulate cerebellar function; however, the absence of similar connectivity patterns in unmedicated siblings and the dose-independent classification performance suggest that medication effects do not fully account for findings. The single-site design and modest sample size limit generalizability. Replication in multi-site consortia is essential, as is prospective validation in clinical high-risk populations and formal integration of fMRI signatures with clinical-cognitive clusters through predictive modeling (Wang et al. (20)).

## 5 Conclusions

This study establishes that schizophrenia is characterized by a reproducible pattern of cerebellum-cortical dysconnectivity and intra-network disorganization, most sensitively detected under moderate working memory load. Independently, unsupervised clustering of clinical-cognitive variables identified data-driven subtypes characterized by distinct cognitive profiles, with cerebellar dysconnectivity showing graded alignment across these subgroups. By integrating multiscale network analysis with data-driven phenotyping, this work demonstrates that neural and clinical heterogeneity in schizophrenia can be characterized through complementary, convergent approaches. These findings support the development of circuit-based stratification tools that leverage task-optimized functional connectivity to enhance biological interpretability beyond traditional diagnostic categories, with potential applications for patient profiling and targeted intervention development.

## Data Availability

Publicly available datasets were analyzed in this study. This data can be found here: https://openneuro.org/datasets/ds000115/versions/00001.
