# Supplementary material for: Cerebellar dysconnectivity in schizophrenia spectrum: task-based functional connectivity analysis and cognitive stratification
Source: Front Psychiatry. 2026 Apr 2;17:1796599. doi: 10.3389/fpsyt.2026.1796599 (PMC13083132; doi:10.3389/fpsyt.2026.1796599)
Supplement: Supplementary file 1 [file DataSheet1.pdf]

Supplementary Material

1 SUPPLEMENTARY TABLES AND FIGURES

Table S1: Preliminary Welch's t-test results comparing schizophrenia patients (SCZ) and healthy controls (CON) across working memory tasks (0-back, 1-back, 2-back). Reported statistics include t-value, uncorrected p-value, FDR-corrected q-value, and Cohen's d effect size. Analyses include inter-network degree, inter-network pairwise connectivity, and intra-network metrics (degree and clustering coefficient).

Task	Metric / Network(s)	t	p	q (FDR)	d
**0-back**	Inter-degree: CER	-0.393	0.6969	0.7665	-0.129
	Inter-degree: CO	-1.179	0.2480	0.5465	-0.408
	Inter-degree: DMN	1.419	0.1655	0.5462	0.479
	Inter-degree: FP	-0.125	0.9011	0.9292	-0.042
	Inter-degree: OCC	1.073	0.2919	0.5465	0.349
	Inter-degree: SM	-1.258	0.2195	0.5465	-0.440
	Inter-pair: CER–CO	-1.910	0.0659	0.4285	-0.659
	Inter-pair: CER–DMN	2.214	0.0342	0.3761	0.726
	Inter-pair: CER–FP	-1.271	0.2149	0.5465	-0.407
	Inter-pair: CER–OCC	1.139	0.2639	0.5465	0.369
	Inter-pair: CER–SM	-3.136	0.0038	0.1260	-1.078
	Inter-pair: CO–DMN	0.740	0.4654	0.6319	0.258
	Inter-pair: CO–FP	-1.064	0.2967	0.5465	-0.372
	Inter-pair: CO–OCC	-0.494	0.6244	0.7105	-0.165
	Inter-pair: CO–SM	-0.975	0.3375	0.5465	-0.336
	Inter-pair: DMN–FP	0.641	0.5262	0.6427	0.218
	Inter-pair: DMN–OCC	1.744	0.0909	0.4285	0.573
	Inter-pair: DMN–SM	0.190	0.8504	0.9053	0.065
	Inter-pair: FP–OCC	0.714	0.4808	0.6319	0.232
	Inter-pair: FP–SM	0.698	0.4919	0.6319	0.247
	Inter-pair: OCC–SM	-1.555	0.1328	0.5317	-0.548
	Intra: CER (degree)	2.329	0.0271	0.3761	0.753
	Intra: CER (clustering)	0.938	0.3551	0.5465	0.314
	Intra: CO (degree)	-1.345	0.1919	0.5465	-0.478
	Intra: CO (clustering)	-0.685	0.4979	0.6319	-0.229
	Intra: DMN (degree)	-0.940	0.3543	0.5465	-0.308
	Intra: DMN (clustering)	-1.855	0.0743	0.4285	-0.597
	Intra: FP (degree)	-1.050	0.3026	0.5465	-0.364
	Intra: FP (clustering)	0.075	0.9410	0.9410	0.025

Continued on next page

Table S1 – continued from previous page

Task	Metric / Network(s)	t	p	q (FDR)	d
	Intra: OCC (degree)	0.921	0.3643	0.5465	0.301
	Intra: OCC (clustering)	0.611	0.5453	0.6427	0.201
	Intra: SM (degree)	-1.771	0.0864	0.4285	-0.604
	Intra: SM (clustering)	-1.496	0.1450	0.5317	-0.513
**1-back**	Inter-degree: CER	-0.767	0.4492	0.6503	-0.250
	Inter-degree: CO	-1.994	0.0546	0.3394	-0.670
	Inter-degree: DMN	1.085	0.2881	0.5003	0.348
	Inter-degree: FP	0.539	0.5933	0.6992	0.179
	Inter-degree: OCC	0.110	0.9130	0.9719	0.036
	Inter-degree: SM	-1.236	0.2251	0.4369	-0.410
	Inter-pair: CER–CO	-3.417	0.0018	0.0298	-1.155
	Inter-pair: CER–DMN	1.553	0.1319	0.3394	0.501
	Inter-pair: CER–FP	-0.660	0.5136	0.6554	-0.220
	Inter-pair: CER–OCC	0.689	0.4967	0.6554	0.222
	Inter-pair: CER–SM	-4.683	0.0000	0.0015	-1.547
	Inter-pair: CO–DMN	0.282	0.7794	0.8869	0.093
	Inter-pair: CO–FP	-1.337	0.1946	0.4013	-0.462
	Inter-pair: CO–OCC	-2.023	0.0523	0.3394	-0.687
	Inter-pair: CO–SM	-0.797	0.4309	0.6503	-0.267
	Inter-pair: DMN–FP	0.656	0.5164	0.6554	0.214
	Inter-pair: DMN–OCC	1.372	0.1835	0.4013	0.438
	Inter-pair: DMN–SM	-0.037	0.9710	0.9733	-0.012
	Inter-pair: FP–OCC	1.592	0.1215	0.3394	0.519
	Inter-pair: FP–SM	1.840	0.0746	0.3394	0.614
	Inter-pair: OCC–SM	-1.576	0.1246	0.3394	-0.528
	Intra: CER (degree)	1.102	0.2782	0.5003	0.365
	Intra: CER (clustering)	-0.034	0.9733	0.9733	-0.011
	Intra: CO (degree)	-1.346	0.1885	0.4013	-0.457
	Intra: CO (clustering)	-1.541	0.1337	0.3394	-0.521
	Intra: DMN (degree)	0.598	0.5551	0.6785	0.192
	Intra: DMN (clustering)	1.561	0.1280	0.3394	0.512
	Intra: FP (degree)	-0.759	0.4532	0.6503	-0.254
	Intra: FP (clustering)	-0.173	0.8641	0.9505	-0.057
	Intra: OCC (degree)	-1.694	0.0997	0.3394	-0.566
	Intra: OCC (clustering)	-0.816	0.4205	0.6503	-0.272
	Intra: SM (degree)	-2.043	0.0490	0.3394	-0.683
	Intra: SM (clustering)	-1.677	0.1029	0.3394	-0.551
**2-back**	Inter-degree: CER	0.288	0.7758	0.8556	0.093
	Inter-degree: CO	-1.601	0.1216	0.4459	-0.549

Continued on next page

Table S1 – continued from previous page

Task	Metric / Network(s)	t	p	q (FDR)	d
	Inter-degree: DMN	0.588	0.5604	0.6956	0.197
	Inter-degree: FP	0.051	0.9594	0.9894	0.017
	Inter-degree: OCC	1.226	0.2298	0.4537	0.399
	Inter-degree: SM	-0.611	0.5461	0.6956	-0.207
	Inter-pair: CER–CO	-1.146	0.2597	0.4692	-0.380
	Inter-pair: CER–DMN	0.634	0.5322	0.6956	0.203
	Inter-pair: CER–FP	-1.375	0.1782	0.4537	-0.454
	Inter-pair: CER–OCC	1.899	0.0707	0.3331	0.605
	Inter-pair: CER–SM	-2.674	0.0126	0.1330	-0.914
	Inter-pair: CO–DMN	-0.577	0.5691	0.6956	-0.198
	Inter-pair: CO–FP	-1.345	0.1883	0.4537	-0.453
	Inter-pair: CO–OCC	-0.373	0.7118	0.8389	-0.124
	Inter-pair: CO–SM	-1.355	0.1870	0.4537	-0.464
	Inter-pair: DMN–FP	0.285	0.7779	0.8556	0.098
	Inter-pair: DMN–OCC	1.300	0.2033	0.4537	0.423
	Inter-pair: DMN–SM	0.002	0.9987	0.9987	0.001
	Inter-pair: FP–OCC	0.728	0.4720	0.6956	0.243
	Inter-pair: FP–SM	1.899	0.0695	0.3331	0.608
	Inter-pair: OCC–SM	-0.648	0.5214	0.6956	-0.216
	Intra: CER (degree)	2.575	0.0161	0.1330	0.828
	Intra: CER (clustering)	1.123	0.2702	0.4692	0.365
	Intra: CO (degree)	-1.049	0.3032	0.5003	-0.357
	Intra: CO (clustering)	-1.497	0.1439	0.4537	-0.500
	Intra: DMN (degree)	-1.701	0.0983	0.4053	-0.569
	Intra: DMN (clustering)	-2.154	0.0385	0.2541	-0.711
	Intra: FP (degree)	-1.238	0.2242	0.4537	-0.412
	Intra: FP (clustering)	-1.213	0.2337	0.4537	-0.398
	Intra: OCC (degree)	0.054	0.9575	0.9894	0.018
	Intra: OCC (clustering)	-0.921	0.3638	0.5716	-0.306
	Intra: SM (degree)	-2.624	0.0151	0.1330	-0.906
	Intra: SM (clustering)	-2.564	0.0151	0.1330	-0.858

Table S2: Influential regions of interest (ROIs) showing significant differences between schizophrenia patients (SCZ) and healthy controls (CON) during working memory tasks (0-back, 1-back, 2-back) at uncorrected $p < 0.05$. Reported statistics include t-value, uncorrected p-value, FDR-corrected q-value, and Cohen's d effect size. Significance markers: $^{*}p < 0.05$, $^{**}p < 0.01$, $^{***}p < 0.001$ (uncorrected).

Task	Network	ROI (Metric)	t	p	q (FDR)	d
**0-back**	CER	Inferior cerebellum (degree)	4.33	0.0000	0.0001	0.525^{***}
	CER	Medial cerebellum (degree)	2.93	0.0038	0.0076	0.391^{**}
	CER	Inferior cerebellum (clustering)	2.80	0.0055	0.0219	0.361^{*}
	OCC	Posterior occipital (degree)	3.95	0.0001	0.0003	0.462^{***}
	SM	Mid insula (degree)	-3.21	0.0019	0.0225	-0.649^{*}
**1-back**	CER	Inferior cerebellum (degree)	4.10	0.0001	0.0002	0.506^{***}
	CER	Inferior cerebellum (clustering)	3.79	0.0002	0.0008	0.481^{***}
	DMN	Angular gyrus (degree)	4.66	0.0000	0.0003	1.073^{***}
	DMN	Precuneus (degree)	4.00	0.0001	0.0008	0.584^{***}
	DMN	Inferior parietal lobule (degree)	3.91	0.0003	0.0014	0.888^{**}
	DMN	Ventromedial prefrontal cortex (degree)	2.89	0.0043	0.0173	0.427^{*}
**2-back**	CER	Inferior cerebellum (degree)	5.74	0.0000	0.0000	0.705^{***}
	CER	Medial cerebellum (degree)	2.51	0.0127	0.0255	0.339^{*}
	CO	Mid insula (clustering)	-3.33	0.0012	0.0229	-0.633^{*}
	CO	Angular gyrus (degree)	3.65	0.0017	0.0320	1.153^{*}
	DMN	Occipital (clustering)	-3.20	0.0017	0.0267	-0.468^{*}
	OCC	Posterior occipital (degree)	3.28	0.0012	0.0035	0.383^{**}
	OCC	Posterior occipital (clustering)	-2.93	0.0036	0.0109	-0.347^{*}
	OCC	Superior frontal (degree)	2.83	0.0063	0.0203	0.648^{*}
	OCC	Posterior cingulate cortex (degree)	2.46	0.0165	0.0441	0.567^{*}
